# Cortical plasticity as a new endpoint measurement for chronic pain

**DOI:** 10.1186/1744-8069-7-54

**Published:** 2011-07-28

**Authors:** Min Zhuo

**Affiliations:** 1 Center for Neuron and Disease, Frontier Institute for Science and Technology, Xian Jiaotong University, Xian, China; 2 Department of Physiology, Faculty of Medicine, University of Toronto Centre for the Study of Pain, Medical Sciences Building, 1 King's College Circle, Toronto, ON, M5S1A8, Canada

## Abstract

Animal models of chronic pain are widely used to investigate basic mechanisms of chronic pain and to evaluate potential novel drugs for treating chronic pain. Among the different criteria used to measure chronic pain, behavioral responses are commonly used as the end point measurements. However, not all chronic pain conditions can be easily measured by behavioral responses such as the headache, phantom pain and pain related to spinal cord injury. Here I propose that cortical indexes, that indicate neuronal plastic changes in pain-related cortical areas, can be used as endpoint measurements for chronic pain. Such cortical indexes are not only useful for those chronic pain conditions where a suitable animal model is lacking, but also serve as additional screening methods for potential drugs to treat chronic pain in humans. These cortical indexes are activity-dependent immediate early genes, electrophysiological identified plastic changes and biochemical assays of signaling proteins. It can be used to evaluate novel analgesic compounds that may act at peripheral or spinal sites. I hope that these new cortical endpoint measurements will facilitate our search for new, and more effective, pain medicines, and help to reduce false lead drug targets.

## Introduction

Pain has at least two different major forms: acute pain that is short-lasting, and often serves as protective and learning signals. It is also called physiological pain in part due to its important physiological functions. The second form is chronic pain that is long-lasting pain caused by injury to tissue or nerve systems. Although it also informs animal or patients the location of the injured area, the long-term component of chronic pain is not physiological critical, and it causes cognitive impairment, emotional sufferings, loss of sleep and mood disorders. Thus, it is also called pathological pain.

**Figure 1:**
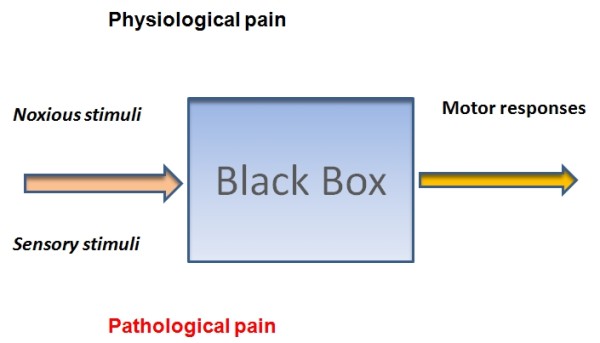
**Black box experimental approaches for the investigation of pain mechanism**. To investigate acute or physiological pain in animals, sensory noxious stimuli are typically used to induce behavioral motor responses in awake or lightly-anesthetized animals. For noxious stimuli, thermal or mechanical stimuli are commonly used. For motor responses, withdrawal reflexes or escaping responses are measured. In some cases, electrodes are used to measure muscle activities (e.g., visceral pain responses). The manipulation or pharmacological agents are introduced to the central nervous systems at different levels (i.e., spinal cord by intrathecal catheters; supraspinal areas by microinjections). Reduction or inhibition of behavioral motor responses is used as the endpoint of pain responses. Complete inhibition of motor responses such as tail-flick or hindpaw withdrawal due to the manipulation in the 'black box' is treated as 100% analgesic or antinociceptive. Similar experimental approaches are often used for studying chronic pain as well in various animal models.

Due to the lack of understanding of cellular and molecular mechanisms of pain mechanisms; the black box experimental approach for studying pain has been widely used by basic researchers and drug developers (Figure 1). Among many different experimental approaches, behavioral reflexes in response to noxious stimuli, or allodynic stimulation after tissue or nerve injury are widely used for the investigation of potential analgesic effects of new drugs, and often proposed as the endpoint of pain indexes. The use of neurobiological approaches is mainly targeted for the investigation of basic mechanisms of pain transmission, modulation and plasticity; and has not been used or proposed as the endpoint measurement of pain.

The progress made in human brain imaging has significantly improved our understanding of chronic pain. Brain imaging in conscious humans allow us to evaluate roles of various cortical areas in pain, and brain activation by painful stimuli can be evaluated and compared with patient's psychological reports of pain and emotional feelings [1]. Recently such techniques have also been successfully used to measure psychological pain or empathy of pain [2,3]. And the measurement of reflexive responses in such human studies is not needed. Can we achieve similar aims in animal studies of chronic pain? In this review, I would like to propose that neurobiological indexes obtained from pain-related cortical neurons can be used as new cortical endpoints of pain measurements in animal models of chronic pain. The use of such new endpoints will allow us to study certain types of chronic pain that is difficult to be investigated in animal models due to the lack of motor responses. They are: headaches, seizure induced pain, phantom pain, spinal injury induced pain and chronic back pain. Furthermore, these new endpoints provide mechanism based measurements of chronic pain in animal models, and could be used as better assays for screening potential new drugs for treating chronic pain. It is important to point out that I am not proposing to use those new endpoints to replace the existing behavioral models of chronic pain such as measuring threshold in hyperalgesia and behavioral responses to allodynic stimulation in chronic pain conditions. The combination of these cortical and behavioral endpoints can provide excellent pain endpoint measurements with solid basic neurobiological mechanisms. The new cortical endpoints can be also used effectively for basic investigation of pain mechanisms at peripheral tissue/nerve, spinal cord, and subcortical areas.

## Problems that we are facing: drug discovery and translational medical researches

While animal models of chronic pain have greatly facilitated our understanding of basic pain mechanisms, there are still many major problems that cannot be solved using animal behavioral models. Many forms of clinical pain cannot be mimicked in animal models. For example, it is very difficult to measure 'phantom' pain in amputated animals; and it is impossible to measure nociceptive withdrawal responses in animals with spinal cord injuries. Mimicking headaches and chronic back pain in animals is also proved to be extremely difficult, since there is not a clear behavioral motor response related to such pain that can be measured. Furthermore, even in many pain conditions that the measurement in animal behavioral responses to pain are possible, the pain index is often affected by unwanted side effects on motor or pre-motor functions. For example, in case of animal models of chemotherapy induced neuropathic pain, the same chemicals also caused injury to motor neurons/nerves. Defects in motor functions directly affect the measurement of nociceptive responses, if only motor responses are used as pain index such as withdrawal thresholds or latencies. Finally, most of potential analgesic drugs also act on motor neurons non-selectively. There is great need to develop additional non-behavioral measurements for chronic pain.

## Human imaging provides direct evidence for the roles of cortex in chronic pain

Unlike animal experiments, human brain imaging using functional magnetic resonance imaging (fMRI) and positron emission tomography (PET) have greatly increased basic neuronal mechanisms for chronic pain, especially at the brain areas. The imaging in conscious patients provides the unique opportunity to investigate if any abnormal neuronal activities may be related to chronic pain in patients [4-7]. Progress made in human imaging of chronic pain is very impressive. Between year 2000 and now, there is an average of 300 papers published per year when the searching key word 'brain imaging', 'pain' and 'human' were used. To deal with this huge amount of imaging data, some mega-analyses have been performed recently [5,8]. Among different cortical regions, there are five major cortical areas that are consistently responding to pain. They are: anterior cingulate cortex (ACC), insular cortex (IC), primary somatosensory cortex (S1), secondary somatosensory cortex (S2) and prefrontal cortex (PFC) (Table 1). Among them, the ACC is found to be the most reliable area to be activated by different noxious or painful stimuli. In addition to the ACC, the IC is also commonly activated by different painful stimuli [9,10,8] for review].

**Table 1:** Cortical areas that are implicated in chronic pain conditions by human brain imaging fMRI that are difficult to be investigated by traditional animal pain models

Cortical area	Clinical pain conditions	Major discovery
*ACC*	Migraine/headache Phantom pain SCI induced pain Chronic back pain	Altered excitatory transmission Related to increased imaged activity Increased activity with imaged pain Increased activity
*IC*	Migraine/headache Chronic back pain Hypothically induced pain	Altered excitatory transmission Increased imaged activity Increased imaged activity
*PFC*	Migraine/headache Phantom pain SCI induced pain Chronic back pain Hypothically induced pain	Altered brain metabolism Related to increased imaged activity Increased activity with imaged pain Correlated with spontaneous pain; reduced gray matter density Increased imaged activity
*S1, S2*	Migraine/headache Phantom pain SCI induced pain	Altered sensory transmission Correlation of cortical reorganization Correlated with cortical reorganization

*Abbreviations:*

ACC: anterior cingulate cortex

IC: insular cortex

PFC: prefrontal cortex

S1: primary somatosensory cortex

S2: Secondary somatosensory cortex

## Classical pain endpoints ---behavioral models of chronic pain

In basic research area, behavioral studies have been commonly used for measuring endpoint of chronic pain. Most of pharmaceutical drug discovery is focused on two major areas: target proteins and the behavioral endpoints, at least to my knowledge. The behavioral evaluations in animal models of chronic pain are used as the end point for preclinical studies. Despite many limitations of behavioral tests (any pre-motor or motor side effects of drugs or gene mutant can easily affect behavioral responses in freely moving conditions), these 'analgesic' indexes are commonly used, because it is economic, fast, and easily understood by laypersons such as private investors.

The failure to provide consistent non-behavioral endpoints for chronic pain also complicates drug discovery; and too many potential lead drugs work well in animal models of chronic pain. In any pain-related conferences held in recent years, you often hear much more positive drug target proteins than negative ones. Furthermore, the failure to require solid basic scientific evidence by the drug regulator encourages the cheap- and short-cut experimental approaches used by private investors and drug developers. Furthermore, many of these 'analgesic drugs' failed to be translated into clinical drugs, in part due to the fact that only behavioral endpoints were used in many of these preclinical studies.

## Clinical image studies of those unstudied chronic pain

It is easy to tell humans are different from animals in term of reporting pain, although humans complain about pain for just for pain. Previous studies reported that animal vocalization activities may be used to measure pain or especially spontaneous pain [11-13]. However, these indexes prove to be difficult to be used, since animals also generate vocalization activities under other normal physiological conditions such as sex mating [11]. Recent human studies using brain imaging have provided key evidence for the involvement of central nervous systems in several chronic pain conditions that cannot be mimicked in animal models. These include headache, phantom pain, back pain, pain related to spinal cord injury, and patient with functional bowel disorder [14,15]. For example, Willoch et al (2000) reported that in human amputees the activity increased in the ACC and posterior cingulate as well as thalamus is correlated with pain reported in phantom limb, while the activity in the supplementary motor cortex and primary sensorimotor cortex is related to the phantom limb movement [16]. Apkarian et al (2004) reported that chronic back pain is associated with decreased prefrontal and thalamic gray matter density [6], and altered neural activity in the PFC [17].

Recent studies of neural correlates of social exclusion by neuroimaging study found that the ACC is more active during exclusion rather than during inclusion [2], suggesting that psychological rejection in social exclusion also triggers pain-related cortical areas. In case of patients with spinal cord injury, it has been reported that the magnitude of activation in the perigenual ACC and right dorsolateral PFC was significantly correlated with absolute increases in pain intensity triggered by movement imagery [18]. Similar findings have also been reported in patients with chronic low back pain [19]. These human studies are in consistent with previous anatomic studies in animals that somatosensory cortex reorganized itself after spinal cord injury [20]. Cumulative human studies data consistently indicate that cortical activity plays an important role in chronic pain. In many cases, changes in cortical activities without any peripheral stimuli (including spontaneous pain conditions) are sufficient to produce pain in patients.

## A proposal for using cortical plasticity as the endpoint measurements for chronic pain

Here I would like to propose the use of cortical plasticity as an endpoint measurement for chronic pain, including biochemical, electrophysiological and imaging measurements (Table 2). Such non-behavioral measurements can also be used to measure cortical responses in animal models that behavioral tests are applicable such as animal models of inflammatory and neuropathic pain. In cases of types of pain where behavioral measurement are impossible, these cortical markers can be used to evaluate pain. Cortical long-term changes can be used as markers for measuring drugs' effects in animal models of chronic pain. Drugs that act at the level of periphery or spinal cord may reduce or inhibit such activation in the cortex if they are potential analgesic for chronic pain.

**Table 2:** Proposed cortical markers for measuring pain endpoints in chronic pain

Category	Measure index
*Immediate early gene*	Activation of c-Fos; Egr1; pCREB
*Cortical potentiation*	Enhanced AMPA receptor mediated EPSCs
	
*Presynaptic enhancement*	
*Paired-pulse facilitation (PPF)*	PPF ratio
*mEPSCs*	Frequency
	Amplitude
*Postsynaptic potentiation*	
*AMPA receptor*	Inward rectification
*NMDA NR2B receptor*	Enhanced NR2B sensitive EPSCs
*PKMζ inhibition*	More sensitive to ZIP inhibition
	
*Biochemistry*	
*AMPA receptor GluR1*	Increased membrane bind AMPA receptor
*p-AMPA receptor*	Increased phosphorylation at PKA site
*PKMζ*	Enhanced pPKMζ
	
*In vivo field LTP*	Injury induced LTP
	
*Structural changes*	Outgrow of neuronal dendrites and increased spine density
	
*Brain imaging & in vivo whole-cell*	Increased spike responses to non-noxious stimuli
	Enhanced cortical activities before and/or after peripheral stimuli

### 1. Activity-dependent immediate early gene (IEG)

Activity-dependent immediate early genes are known to be activated by neuronal activity in the central nervous system. Peripheral noxious stimuli or injury triggered activation of c-Fos and phosphorylation of cAMP response element binding protein (pCREB) in the spinal cord dorsal horn neurons [21,8]. Such activation in the spinal cord dorsal horn neurons can be inhibited or blocked by drugs or inhibited behavioral sensitization. It is believed that these activity-activated immediate early genes may contribute to long-term plastic changes in spinal sensory synapses, and thus contribute to behavioral sensitization. It is expected that analgesics that acts at the level of spinal cord or periphery for the treatment of chronic pain should at least partially reduce activation of IEGs triggered by the injury.

**Figure 2:**
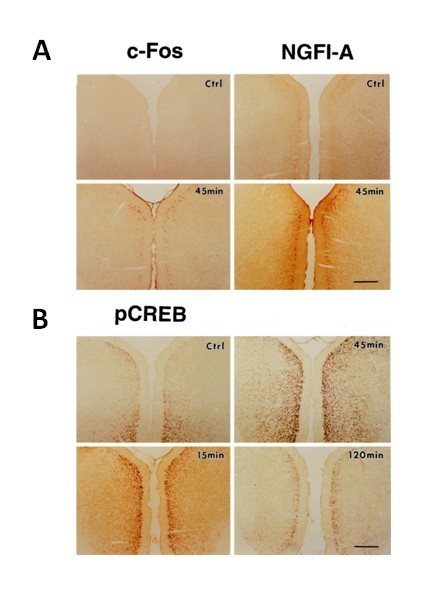
**Potentiation of IEG expression in the ACC after the amputation**. Photomicrographs showing expression of c-Fos and NGFI-A (A) and phosphorylation of CREB (B) in the coronal ACC sections from sham animals (Ctrl) and animals at different times after the amputation of the unilateral hindpaw third digit. Scale bars, 500 μm. The numbers of c-Fos-, NGFI-A-, and pCREB-immunoreactive cells increased bilaterally after the amputation (from Reference 18).

Activation of IEGs is not limited at the spinal cord level. Along the somatosensory pathways, activation of IEGS have also been reported in the thalamus, and related cortical areas. Among several pain-related cortical areas, activation of IEGs in cortical neurons after the injury has been reported in the ACC and IC. Using c-Fos, NGFI-A (also called Egr1, zif268), and pCREB, neurons in the ACC are known to be activated by peripheral noxious stimuli, injury or amputation [22-24]. Wei et al (1999) reported that digit amputations in adult rats triggered rapid activation of Fos, Egr1 and CREB in the ACC neurons, especially pyramidal cells located in the layer II-III (Figure 2). Activation of IEGs is bilateral. It persists for two days [23]. Similar activation of IEGs in the ACC has been reported after tissue inflammation, nerve injury and visceral pain [8].

Using gene knockout mice, it has been shown that the amount of IEG activation may be related to behavioral pain phenotypes. In adenylyl cyclase subtype 1 (AC1) or AC1 and AC8 knockout mice, it has been shown that chronic inflammatory pain and neuropathic pain were significantly reduced. Consistently, pCREB immunoreactivity induced by hindpaw inflammation was also reduced in AC1, AC8, or AC1&8 double knockout (DKO) mice. Activation of IEGs is also noted in other cortical areas such as IC [25]. Similar to the ACC, the amount of gene expression is likely also related to behavioral pain phenotypes. Interestingly, fear condition, a form of emotional learning, also triggered IEGs in cortical neurons [26]. Activation of Ca2+-calmodulin-dependent protein kinase IV (CaMKIV) is functionally important for activation of IEGs as well as behavioral memory [26]; indicating that different intracellular signaling pathways may be involved in mediating fear and chronic pain.

**Figure 3:**
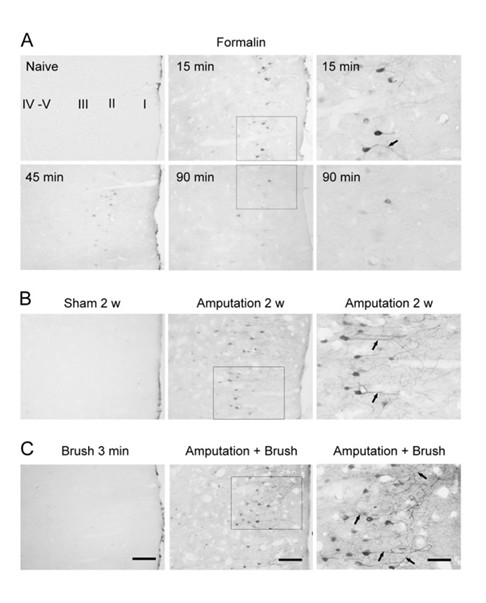
**Enhanced Erk activation in the ACC after tissue and nerve injury**. A. Immunohistochemical staining for phosphorylation of Erk illustrated time course-dependent activation of Erk in layer II neurons of the contralateral ACC after unilateral hindpaw injection of formalin (5%, 50 μl, n = 4-5 rats for each time point). B. The P-Erk expression in the layer II ACC neurons and their main apical dendrites (arrows) was increased at 2 weeks after the amputation of the unilateral hindpaw third digit (n = 5), compared to sham animals (n = 3). C. Mechanical stimulation by brushing hindpaw of digit amputation induced P-Erk expression in more number of layer II ACC neurons and the more distinctive apical dendrites at 2 weeks after the amputation (n = 5), compared to that in rats with amputation alone. There was not P-Erk activation in the ACC in normal animals after the brushing (n = 3). Left and middle columns: low power of the coronal ACC sections. Scale bar = 50 μm; Right column: enlarged layer II regions corresponding to the small rectangle areas in the middle column, respectively. Scale bar = 25 μm (from Reference 22).

In addition to assay baseline neuronal changes after the injury, some of these makers can be also used to evaluate allodynic responses in animals with the injury. In animals at two weeks after the injury, most of IEGs activities return to baseline level in the ACC. Non-noxious stimuli that usually did not induce any obvious behavioral response and gene expression in the ACC triggered remarkable IEG activation within the ACC [see [27]] (Figure 3). Similar studies in adult rats with nerve injury have reported that mild noxious stimuli triggered significant activation of phosphorylated-c-Jun activity in the cingulate cortex [28].

### 2. Cortical potentiation

**Figure 4:**
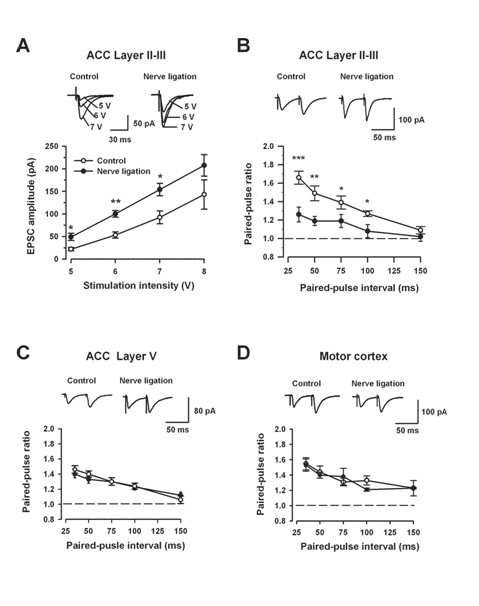
**Increased synaptic transmission in layer II/III in the ACC after peripheral nerve ligation**. A, Synaptic input-output curves in slices from control (n = 6 neurons) and nerve-ligated (n = 7 neurons) mice. *p < 0.05 and **p < 0.01 compared with those of control group. Open circles, Neurons from control mice; filled circles, neurons from mice with nerve ligation. B, Representative traces with an interval of 50 ms recorded in layer II/III of the ACC. Paired-pulse ratio (the ratio of EPSC2/EPSC1) was recorded at intervals of 35, 50, 75, 100, and 150 ms from control and nerve-ligated mice. Open circles, Neurons from control mice (n = 17 neurons); filled circles, neurons from mice with nerve ligation (n = 19 neurons). *p < 0.05; **p < 0.01; ***p < 0.001. C, PPF in layer V of the ACC from control and nerve-ligated mice. Open circles, Neurons from control mice (n = 9 neurons); filled circles, neurons from mice with nerve ligation (n = 15 neurons). D, PPF in motor cortex neurons from control and nerve-ligated mice. Open circles, Neurons from control mice (n = 5 neurons); filled circles, neurons from mice with nerve ligation (n = 5 neurons) (from reference 31).

Glutamate is the major excitatory fast transmitter in the cortical areas including the ACC [29]. Postsynaptic synaptic responses are mainly mediated by postsynaptic glutamate α-amino-3-hydroxy-5-methyl-4-isoxazole-propionate (AMPA) receptors with a small portion of currents are mediated by kainate (KA) receptors [29]. It has been proposed that excitatory synapses in the ACC undergo long-term potentiation (LTP) after peripheral injury [21,8]. Considering ACC neurons show wide spread activation to peripheral injury [22], it is much easier to detect changes in excitatory synaptic transmission in the ACC area. To explore whether there is any change in basal synaptic transmission within the ACC after nerve injury, AMPA receptor-mediated EPSCs in pyramidal neurons in the layer II/III of the ACC are measured in mice with peripheral nerve ligation [30]. Recorded neurons were identified as pyramidal neurons based on their ability to show spike frequency adaptation in response to the prolonged depolarizing-current injection [31-33]. Indeed, we found that input (stimulation intensity)-output (EPSC amplitude) curve of AMPA receptor-mediated current was significantly shifted to the left after peripheral nerve injury, compared with that in control group (Figure 4). These results suggest that excitatory synaptic transmission was increased in the ACC after peripheral nerve injury.

Similar changes are found in ACC neurons in animal models of inflammation induced by complete Freund's adjuvant (CFA) [34]. CFA injection caused significant potentiation of the input-output relationship of the glutamate mediated excitatory transmission in the ACC of adult mice. Similar changes were found in animals with inflammation in rats (CFA model). Bie et al (2010) reported that CFA inflammation in rats triggered increased AMPA receptor mediated responses in ACC neurons with observed leftward shift of the input-out curves [35].

### 3. Presynaptic enhancement of glutamate release

Paired-pulse facilitation (PPF) is a transient form of plasticity commonly used as a measure of presynaptic function, in which the response to the second stimulus is enhanced as a result of residual calcium in the presynaptic terminal after the first stimulus [34,36]. In control mice, PPF was observed at different stimulus intervals of 35, 50, 75, 100, and 150 ms. After nerve ligation, there was a significant reduction in PPF in ACC neurons compared with those from control mice. The changes in PPF ratio is selective for neurons in layer II/III, no obvious changes were detected in deeper neurons in the ACC. These results indicate that presynaptic enhancement of the excitatory synaptic transmission selectively occurs in the layer II/III of the ACC after nerve injury (Figure 4). Similar changes in PPF ratio are found in ACC neurons of animals with CFA inflammation [33], indicating that presynaptic enhancement of glutamate release is also shared by peripheral inflammation.

In addition to the use of PPF in the ACC after peripheral nerve injury, AMPA receptor-mediated miniature excitatory postsynaptic currents (mEPSCs) in ACC neurons in the presence of 0.5 μM tetrodotoxin (TTX) were also found to be affected. After peripheral nerve injury, there was an obvious increase of mEPSC frequency in ACC neurons compared with that of control group. Furthermore, there was significant difference in the amplitude of mEPSCs between the two groups, indicating postsynaptic changes in AMPA receptor mediated responses (see below). By contrast, no significant change in mEPSCs amplitudes is detected in animal model of inflammation [34]. However, this does not completely rule out possible postsynaptic changes that may contribute to inflammatory pain in the ACC.

In a recent study, the use of c-Fos transgenic mice allows one to selectively record pain-trigger cortical cells [37]. The combination of c-Fos transgenic mice and PPF can help to detect selective changes in pain-activated synapses.

### 4. Postsynaptic glutamate mediated responses

#### AMPA receptor

AMPA receptors without GluR2 are Ca^2+ ^permeable and inwardly rectifying [38-40]. Inward rectification occurs by voltage-dependent blockade by polyamines [41]. To identify whether there are inwardly rectifying properties of AMPA receptors as a result of an alteration of their subunit composition in ACC neurons after nerve injury, AMPA receptor-mediated EPSCs were induced at the holding potentials of -65, -5, and +35 mV in ACC neurons. We found that there was significant difference in the rectification of AMPA receptor-mediated transmission in the ACC between control and nerve-ligated mice [36]. Consistently, when the mean current-voltage (I-V) relationship was plotted, less inward currents were found in ACC neurons from mice with nerve injury compared with control mice [36]. These results demonstrate that AMPA receptor in ACC neurons has an inward rectification property in neuropathic pain.

Similar rectification of the AMPA receptor mediated responses in ACC neurons of rats has been reported after peripheral inflammation with hindpaw CFA injection [35].

#### Postsynaptic NMDA NR2B receptor

**Figure 5:**
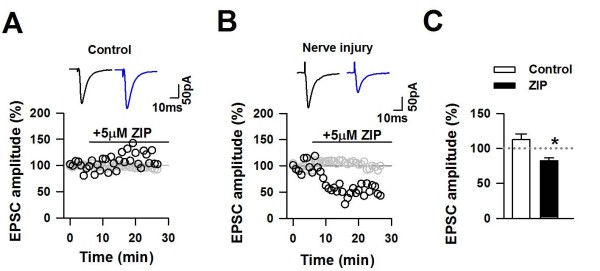
**Increased NMDA NR2B receptor-mediated postsynaptic currents in adult ACC neurons is shown after inflammation**. A, Confocal image of a pyramidal neuron in an adult ACC slice loaded with Lucifer yellow (top left). The arrow indicates the location of the neuron in the ACC slice (bottom left). Scale bar, 20 μm. When injected with current steps from -100 to 100 pA in 400 ms (bottom right), the neuron shown at the left fired repetitive action potentials with frequency adaptation (top right). A minority of neurons recorded (n = 4) showed the fast-spike properties (middle right). B, Current-voltage plot of the NMDA receptor EPSCs. The currents exhibited strong outward rectification and the reversal potential of the linear part was 4.4 ± 2.0 mV (n = 6 neurons/5 mice). Inset, NMDA receptor-mediated EPSCs recorded at holding potentials from -65 to 50 mV. Each trace represents the average of five consecutive recordings. C, A selective NR2B antagonist, Ro 25-6981, partially inhibited NMDA receptor-mediated EPSCs. The time course of changes in EPSC amplitude before and during the application of Ro 25-6981 (0.3 and 3 μM) and AP-5 (50 μM) in ACC neurons from both saline (control, Δ) and CFA-injected () mice is shown. Traces show the currents at different time points during application of drugs. Ro 25-6981 produced its maximal effect at 3 min after bath application, and a higher dose of Ro 25-6981 (3 μM) had no additional effects. The remaining currents can be totally blocked by AP-5 (50 μM). D, Summary data of the effects Ro 25-6981 (0.3 and 3 μM) in ACC neurons of control (saline-injected) and CFA-injected mice. Ro 25-6981 produced significantly greater inhibitory effects on NMDA receptor-mediated EPSCs in ACC neurons in CFA-injected mice (n = 9 neurons/8 mice) than those in control adult mice (n = 8 neurons/7 mice). AP-5 (50 μM) completely blocked the currents, confirming the currents are mediated NMDA receptors (n = 5 slices/5 mice). The double asterisks indicate significant difference from control, and the double daggers indicate significant difference from control NMDA receptor-mediated EPSCs. Error bars represent SEM (from Reference 24).

Evidence for the involvement of cortical NMDA NR2B receptor in chronic pain first comes from the NMDA NB2B forebrain overexpressed 'smart' mice [42,43]. In this transgenic mouse with selective forebrain NMDA NB2B overexpression, inflammatory pain and allodynia were significantly enhanced without any significant effect on acute pain. Subsequent studies demonstrated that peripheral inflammation with CFA injection triggered long-lasting increases in the expression of NMDA NR2B receptor proteins in the ACC [44,45]. The changes in NMDA receptor protein expression are subtype selective, since other NMDA receptor subunits such as NR1 and NR2A did not show any significant change (Figure 5). The increased NR2B receptors are likely to be within synapses, because single-shock focal stimulation induced NR2B receptor-mediated synaptic currents were enhanced. Furthermore, a recent study reported that gentiopicroside, an analgesic compound for inflammatory pain, reduced the expression of ACC NR2B induced by the injury, as well as NMDA receptor mediated responses [45].

In an animal model of arthritic pain, it has been reported that inflammation increased phosphorylation but not up-regulation of NMDA NR1 proteins in the central amygdale [46]. In parallel, NMDA receptor mediated responses are also enhanced.

#### Sensitivity to inhibition by protein kinase M zeta (PKMζ) inhibitor ZIP

**Figure 6:**
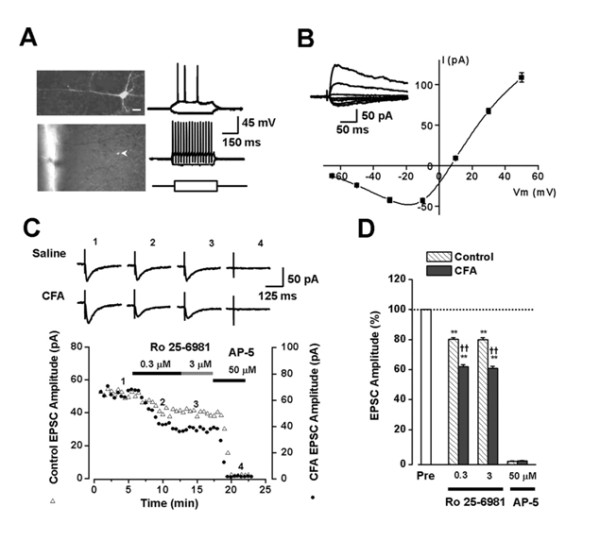
**Inhibition of PKMζ selectively decreased the amplitude of eEPSCs in ACC neurons of mice with neuropathic pain by reducing the number of active AMPA receptors**. A and B, Samples showed the effect of ZIP (5 μM) on the amplitude of eEPSCs in the ACC neurons of animals from the nerve injury [black circles in (A)] and sham group [black circles in (B)]. The gray open circles represent the change of membrane resistance during recording. Black traces in the upper part of (A) and (B) indicate the averaged response at baseline, and blue traces indicate the average of 2 min responses collected 10 min after ZIP application. C, Pooled data of effects of ZIP on the eEPSCs recorded from the ACC of mice in the sham (open) and nerve injury (solid) groups 10 min after ZIP application. *P < 0.05; error bars, SEMs (from Reference 32).

What is likely to be the synaptic mechanism responsible for the analgesic effects produced by ZIP in neuropathic pain? PKMζ can potentiate postsynaptically the amplitude of AMPA receptor-mediated EPSCs [37,47]. Because glutamatergic synaptic transmission in the ACC is increased after nerve injury [36], we speculated that PKMζ may contribute to the maintenance of enhanced synaptic transmission induced by nerve injury. First, we recorded AMPA receptor-mediated EPSCs in layer II/III of the ACC 3 or 7 days after nerve injury [37]. We found that bath application of ZIP (5 μM) significantly reduced eEPSCs (Figure 6). In contrast, ZIP did not affect the amplitude of eEPSCs recorded in neurons from sham-operated mice.

### 5. Biochemical markers

#### Membrane GluR1 expression

The trafficking of AMPA receptor subunits has been proposed to contribute to synaptic plasticity underlying hyperalgesia [48-50]. We investigated the distribution of AMPA receptor subunits in the ACC after nerve ligation. We found that induction of neuropathic pain by nerve ligation was associated with an increase in the abundance of the GluR1 subunits in the membrane fraction and a corresponding decrease in the levels in the cytosolic fraction. In contrast, nerve ligation had no effect on the intracellular distribution of GluR2/3 subunits in ACC neurons [36]. The data show that AMPA receptor GluR1 subunit is redistributed in ACC neurons as a result of nerve injury.

In rats with CFA inflammation, Bie et al reported that AMPA GluR1 in the synapsome preparation of the ACC neurons from rats with CFA injection showed significant increases, suggesting that synaptic AMPA GluR1 is significantly increased [35].

#### Phosphorylation of GluR1

The phosphorylation of GluR1 subunit of AMPA receptors is critical for synaptic expression of the receptors, their channel properties, and synaptic plasticity [51-53]. We tested the phosphorylation levels of GluR1 subunit at the PKA phosphorylation site (Ser 845) in the ACC of the mice with nerve ligation. We found that the phosphorylation levels of GluR1 were significantly increased in the ACC after nerve injury. The data indicate that the nerve injury can increase the phosphorylation levels of GluR1 through the PKA signaling pathway.

#### Upregulation of PKMζ as an marker

**Figure 7:**
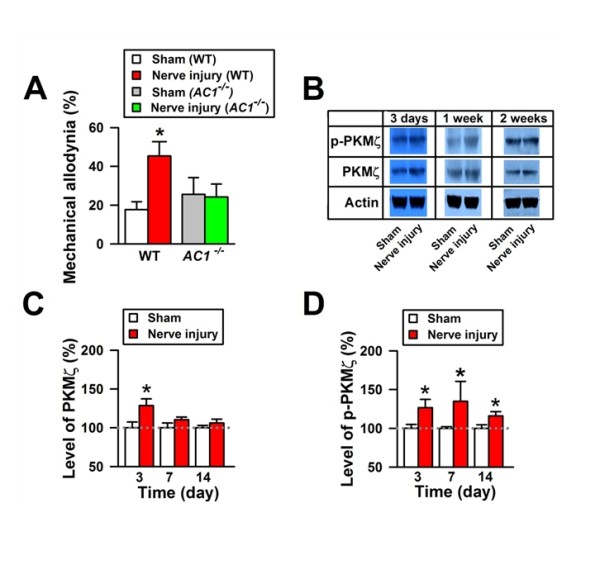
**Expression and phosphorylation of PKMζ in the ACC during neuropathic pain**. A, Mechanical allodynia was tested in the sham and nerve injury groups from wild-type (WT) and AC1^-/- ^animals 3 days after nerve injury. * indicates P < 0.05; error bars indicate SEMs. B, Western blots for PKMζ and p-PKMζ in the ACC obtained between 3 days and 2 weeks after nerve injury. (The ACC of mice from the sham and nerve injury groups were used for Western blot analysis 90 min after the allodynia test.) C, Level of PKMζ in the ACC of mice from the sham and nerve injury groups. Level of PKMζ increased significantly 3 days after nerve injury compared with PKMζ in the sham group. D, Level of p-PKMζ increased significantly 3 days after nerve injury. This effect lasted over 2 weeks after nerve injury compared with p-PKMζ in the sham group (from Reference 32).

We examined whether, in the ACC, peripheral nerve injury causes changes in PKMζ [37]. Behavioral allodynic response was increased 3 days after nerve injury when compared with the response in sham-treated mice. The levels of PKMζ in the ACC were significantly increased after nerve injury. Because PKMζ is activated by phosphorylation, we also conducted experiments to detect possible changes in the level of phosphorylated PKMζ (p-PKMζ). Consistently, the level of p-PKMζ was also significantly increased. These data suggest that peripheral nerve injury increases PKMζ activity in the ACC. To determine whether such changes are long-lasting, we examined PKMζ and p-PKMζ levels 7 and 14 days after nerve injury. Although behavioral allodynia persisted at these time points, the protein levels of PKMζ returned to baseline (Figure 7), indicating that the regulation of the amount of PKMζ is short-lasting. However, the p-PKMζ level remained increased, suggesting that PKMζ activity could contribute to the maintenance of neuropathic pain. To investigate whether the changes in PKMζ protein levels or activity were a generalized phenomenon in the central nervous system, we also examined the levels of PKMζ and p-PKMζ in the hippocampus and spinal dorsal horn 3 days after nerve injury.

### 6. In vivo electrophysiological as direct evidence for chronic pain

**Figure 8:**
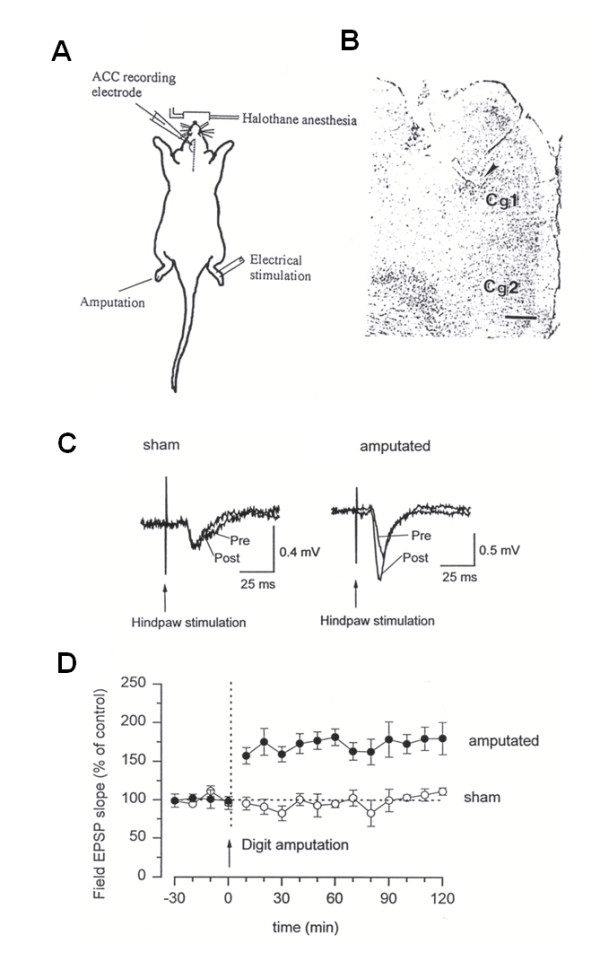
**Sensory response of the anterior cingulate cortex to peripheral stimulation in adult rats**. A-B, diagram of in vivo recording from the anterior cingulate cortex (ACC) in an anaesthetized rat; animals were maintained in a lightly anaesthetized state by halothane. The recording electrode was placed into the ACC contralateral to the peripheral stimulation electrode. Amputation (the removal of the third digit of the hindpaw) was performed on the non-stimulated hindpaw. During amputation, a higher concentration of halothane was used. C, Long-lasting enhancement following amputation of a single hindpaw digit A, representative traces of EPSPs 5 min before amputation (Pre) and 115-120 min after (Post) sham treatment or amputation. The he latency of sensory responses was not changed after the amputation, while the EPSP slope was increased. D, amputation of a single digit of the contralateral hindpaw (indicated by an arrow) caused long-lasting enhancement of sensory responses (•). Sensory responses were not significantly changed in sham-treated animals (○). The test stimulation frequency was 0.01 Hz (from Reference 54).

In brain slices, cingulate synapses can undergo LTP after experimentally designed training protocols. One key question regarding ACC plasticity is whether or not injury causes long-term changes in synaptic transmission in the ACC in intact animals. To test this, we performed experiments in anesthetized rats. We measured synaptic responses to peripheral electrical shocks by placing a recording electrode in the ACC of anesthetized rats [54]. At high intensities of stimulation, sufficient to activate nociceptive A_δ _and C fibers, evoked field EPSPs were recorded in the ACC. Digit amputation at the contralateral hindpaw causes a rapid and long-lasting enhancement (more than 120 min) of sensory responses (Figure 8). Potentiated sensory responses do not require persistent activity from the injured hindpaw [54]. These findings indicate that plastic changes are likely occurring within the ACC synapses. Furthermore, in vivo intracellular recordings from anesthetized rats have confirmed similar findings [55].

### 7. Long-term structural changes

It has been known that learning related plasticity requires transcriptional and translational processing and triggers long-term structural changes in individual synapses [56]. Considering wide-spreading neuronal plasticity happens in the central nervous system after a peripheral injury, it is expected to find long-term changes in brain areas related to chronic pain. The well known cortical reorganization of somatosensory cortex was reported in monkeys with amputated arms. Recently, in patients with chronic pain such as chronic back pain, loss of cortical areas has been reported [7,57].

In animal models of inflammatory pain or chronic pain, it has been reported that inhibiting macromolecular synthesis is analgesic [58]. Structural or synaptic changes after nerve injury have also been reported in cortical areas [59,60]. Metz et al reported that layer 2/3 pyramidal neurons in acute slices of the contralateral medial prefrontal cortex (mPFC) in the rat spared nerve injury model of neuropathic pain showed morphological differences between the mPFC of injured and sham-operated animals [60]. Basal dendrites of neurons from injured rats are longer and have more branches than their counterparts in sham-operated animals; spine density is also selectively increased in basal dendrites of neurons from injured rats.

### 8. Imaging ACC activity and spike recording in experimental animals

Electrophysiological recordings of sensory induced unit responses from cortical neurons is another direct measure of injury induced cortical changes. The use of responsive threshold, the measurement of receptive field, and changes in the magnitude of nociceptive responses are good indexes for injury related cortical plasticity. It is likely that heterogeneous populations of cells are likely found. For example, in the amygdala, Neugebauer and Li reported (2002) that amygdala neurons showed differential sensitization (measured by spike activities) to sensory afferent inputs in a model of arthritic pain [61].

In addition to spike recordings, recent studies using whole-cell patch-clamp recordings from adult ACC neurons found that many of ACC neurons are responsive to peripheral nociceptive stimulation. Whole-cell patch-clamp recording offer better sensitivity for detecting possible changes in chronic pain, and evaluate the possible analgesic effects of drugs [62].

Recent several studies have reported brain activation in awake rats using the imaging technique [63]. In this novel approach, Becerra et al (2010) performed fMRI in trained, acclimated, awake rats [63]. The new approach avoids the potential complicating effects of anesthesia. Differing from experiments in humans, animal needs to be kept under anesthesia state to avoid the movement. Among many cortical areas, they reported that ACC, somatosensory cortexes and IC are activated. This approach can be effectively used for evaluating effects of new drugs on pain in awake animals.

## Examples how cortical endpoints can be used

It is known that injury triggers molecular and cellular changes in different parts of the brains. Depending on the specific regions of the brain, these neurobiological changes may contribute to different aspects of pain, such as learning and memory, anxiety, unpleasantness and attention. Although some of these changes can be observed at behavioral levels (i.e., reduced latency to withdrawal; or enhanced responses to noxious stimuli), some are difficult to be studied at behavioral level. One good example to use the cortical endpoints is to evaluate the effects of new drugs in spinal cord injury induced pain. It is commonly reported in patients with spinal cord injury that they suffer long-lasting neuropathic pain caused by the injury. However, in animal models of spinal cord injury, it is impossible to evaluate the drug's analgesic effect using behavioral tests. Using the proposed cortical endpoints, I propose that these cortical endpoints can be used. For example, activation of various IEGs in the pain-related cortex such as ACC can be used to evaluate early activation, and measuring glutamate mediated responses and biochemical markers can be used to evaluate enhanced transmission during chronic pain induced by spinal cord injury. The drugs that inhibited or erased cortical makers may be potentially useful for treating spinal cord injury related chronic pain in patients.

The same endpoints can be used to evaluate drugs that acting at peripheral or spinal mechanisms. In additional behavioral responses, cortical endpoints can be measured before and after drug application. Drugs that reduce peripheral or spinal sensitization or potentiation should reduce activation of cortical markers. The cortical measurement will help to determine if behavioral inhibition is due to pure inhibition of motor neurons that are required for sensitized withdrawal responses in chronic pain conditions. There are some limitations of using these proposed cortical indexes to study 'pain' in animals. It is important to perform additional experiments to distinguish possible changes in these cortical indexes that are important for pain-related memory but not pain itself. Considering that cortical neurons are often activated in different situations, it is not an easy task if the injury is also located within the brain. The use of selective gene-manipulated mice or pharmacological inhibitors may help to address some of these concerns.

## Conclusion

In summary, recent basic neurobiological investigations of physiological and pathological mechanisms of pain and chronic pain provide critical information for our understanding of long-term plastic changes in cortical areas. These cortical changes that persisted during the course of chronic pain can be used a valuable index to measure 'pain' in animals. The combined use of these neurobiological indexes, together with or without behavioral motor withdrawal responses, will greatly facilitate our searching for new drugs, and also help us to understand why some of current pain medicine does not work in some clinical conditions.

## Competing interests

The author declares that they have no competing interests.

## Authors' contributions

MZ designed and finished the manuscript.

## References

[B1] Talbot JD Marrett S Evans AC Meyer E Bushnell MC Duncan GH Multiple representations of pain in human cerebral cortex Science 1991 251 1355 1358 10.1126/science.2003220 2003220

[B2] Eisenberger NI Lieberman MD Williams KD Does rejection hurt? An FMRI study of social exclusion Science 2003 302 290 292 10.1126/science.1089134 14551436

[B3] Singer T Seymour B O'Doherty J Kaube H Dolan RJ Frith CD Empathy for pain involves the affective but not sensory components of pain Science 2004 303 1157 1162 10.1126/science.1093535 14976305

[B4] Hofbauer RK Olausson HW Bushnell MC Thermal and tactile sensory deficits and allodynia in a nerve-injured patient: a multimodal psychophysical and functional magnetic resonance imaging study Clin J Pain 2006 22 104 108 10.1097/01.ajp.0000149798.93498.7c 16340599

[B5] Apkarian AV Bushnell MC Treede RD Zubieta JK Human brain mechanisms of pain perception and regulation in health and disease Eur J Pain 2005 9 463 484 10.1016/j.ejpain.2004.11.001 15979027

[B6] Apkarian AV Sosa Y Sonty S Levy RM Harden RN Parrish TB Gitelman DR Chronic back pain is associated with decreased prefrontal and thalamic gray matter density J Neurosci 2004 24 10410 10415 10.1523/JNEUROSCI.2541-04.2004 15548656 PMC6730296

[B7] Flor H Nikolajsen L Staehelin Jensen T Phantom limb pain: a case of maladaptive CNS plasticity? Nat Rev Neurosci 2006 7 873 881 17053811 10.1038/nrn1991

[B8] Zhuo M Cortical excitation and chronic pain Trends Neurosci 2008 31 199 207 10.1016/j.tins.2008.01.003 18329111

[B9] Brooks JC Zambreanu L Godinez A Craig AD Tracey I Somatotopic organisation of the human insula to painful heat studied with high resolution functional imaging Neuroimage 2005 27 201 209 10.1016/j.neuroimage.2005.03.041 15921935

[B10] Schreckenberger M Siessmeier T Viertmann A Landvogt C Buchholz HG Rolke R Treede RD Bartenstein P Birklein F The unpleasantness of tonic pain is encoded by the insular cortex Neurology 2005 64 1175 1183 10.1212/01.WNL.0000156353.17305.52 15824343

[B11] Ko SW Chatila T Zhuo M Contribution of CaMKIV to injury and fear-induced ultrasonic vocalizations in adult mice Mol Pain 2005 1 10 10.1186/1744-8069-1-10 15813959 PMC1079936

[B12] Martino G Perkins MN Tactile-induced ultrasonic vocalization in the rat: a novel assay to assess anti-migraine therapies in vivo Cephalalgia 2008 28 723 733 10.1111/j.1468-2982.2008.01582.x 18498397

[B13] Fu Y Neugebauer V Differential mechanisms of CRF1 and CRF2 receptor functions in the amygdala in pain-related synaptic facilitation and behavior J Neurosci 2008 28 3861 3876 10.1523/JNEUROSCI.0227-08.2008 18400885 PMC2557030

[B14] Kwan CL Diamant NE Pope G Mikula K Mikulis DJ Davis KD Abnormal forebrain activity in functional bowel disorder patients with chronic pain Neurology 2005 65 1268 1277 10.1212/01.wnl.0000180971.95473.cc 16247056

[B15] Prescot A Becerra L Pendse G Tully S Jensen E Hargreaves R Renshaw P Burstein R Borsook D Excitatory neurotransmitters in brain regions in interictal migraine patients Mol Pain 2009 5 34 10.1186/1744-8069-5-34 19566960 PMC2714306

[B16] Willoch F Rosen G Tolle TR Oye I Wester HJ Berner N Schwaiger M Bartenstein P Phantom limb pain in the human brain: unraveling neural circuitries of phantom limb sensations using positron emission tomography Ann Neurol 2000 48 842 849 10.1002/1531-8249(200012)48:6<842::AID-ANA4>3.0.CO;2-T 11117540

[B17] Baliki MN Geha PY Jabakhanji R Harden N Schnitzer TJ Apkarian AV A preliminary fMRI study of analgesic treatment in chronic back pain and knee osteoarthritis Mol Pain 2008 4 47 10.1186/1744-8069-4-47 18950528 PMC2584040

[B18] Gustin SM Wrigley PJ Henderson LA Siddall PJ Brain circuitry underlying pain in response to imagined movement in people with spinal cord injury Pain 148 438 445 10.1016/j.pain.2009.12.001 20092946

[B19] Kobayashi Y Kurata J Sekiguchi M Kokubun M Akaishizawa T Chiba Y Konno S Kikuchi S Augmented cerebral activation by lumbar mechanical stimulus in chronic low back pain patients: an FMRI study Spine (Phila Pa 1976) 2009 34 2431 2436 10.1097/BRS.0b013e3181b1fb76 19789470

[B20] Jain N Florence SL Kaas JH Reorganization of Somatosensory Cortex After Nerve and Spinal Cord Injury News Physiol Sci 1998 13 143 149 11390778 10.1152/physiologyonline.1998.13.3.143

[B21] Zhuo M Neuronal mechanism for neuropathic pain Mol Pain 2007 4 14 10.1186/1744-8069-3-14 PMC1904186 17553143

[B22] Ko SW Vadakkan KI Ao H Gallitano-Mendel A Wei F Milbrandt J Zhuo M Selective contribution of Egr1 (zif/268) to persistent inflammatory pain J Pain 2005 6 12 20 10.1016/j.jpain.2004.10.001 15629414

[B23] Wei F Li P Zhuo M Loss of synaptic depression in mammalian anterior cingulate cortex after amputation J Neurosci 1999 19 9346 9354 10531439 10.1523/JNEUROSCI.19-21-09346.1999 PMC6782899

[B24] Wei F Xu ZC Qu Z Milbrandt J Zhuo M Role of EGR1 in hippocampal synaptic enhancement induced by tetanic stimulation and amputation J Cell Biol 2000 149 1325 1334 10.1083/jcb.149.7.1325 10871275 PMC2175137

[B25] Wei F Qiu CS Liauw J Robinson DA Ho N Chatila T Zhuo M Calcium calmodulin-dependent protein kinase IV is required for fear memory Nat Neurosci 2002 5 573 579 10.1038/nn0602-855 12006982

[B26] Wei F Qiu CS Kim SJ Muglia L Maas JW Pineda VV Xu HM Chen ZF Storm DR Muglia LJ Zhuo M Genetic elimination of behavioral sensitization in mice lacking calmodulin-stimulated adenylyl cyclases Neuron 2002 36 713 726 10.1016/S0896-6273(02)01019-X 12441059

[B27] Wei F Zhuo M Activation of Erk in the anterior cingulate cortex during the induction and expression of chronic pain Mol Pain 2008 4 28 10.1186/1744-8069-4-28 18651976 PMC2503974

[B28] Niikura K Furuya M Narita M Torigoe K Kobayashi Y Takemura Y Yamazaki M Horiuchi H Enomoto T Iseki M Kinoshita H Tomiyasu S Imai S Kuzumaki N Suzuki T Enhancement of glutamatergic transmission in the cingulate cortex in response to mild noxious stimuli under a neuropathic pain-like state Synapse858:38 65 424 432 10.1002/syn.20859 20812294

[B29] Wu LJ Zhao MG Toyoda H Ko SW Zhuo M Kainate receptor-mediated synaptic transmission in the adult anterior cingulate cortex J Neurophysiol 2005 94 1805 1813 10.1152/jn.00091.2005 15928066

[B30] Vadakkan KI Jia YH Zhuo M A behavioral model of neuropathic pain induced by ligation of the common peroneal nerve in mice J Pain 2005 6 747 756 10.1016/j.jpain.2005.07.005 16275599

[B31] Tsvetkov E Shin RM Bolshakov VY Glutamate uptake determines pathway specificity of long-term potentiation in the neural circuitry of fear conditioning Neuron 2004 41 139 151 10.1016/S0896-6273(03)00800-6 14715141

[B32] Zhao MG Toyoda H Lee YS Wu LJ Ko SW Zhang XH Jia Y Shum F Xu H Li BM Kaang BK Zhuo M Roles of NMDA NR2B subtype receptor in prefrontal long-term potentiation and contextual fear memory Neuron 2005 47 859 872 10.1016/j.neuron.2005.08.014 16157280

[B33] Cao XY Xu H Wu LJ Li XY Chen T Zhuo M Characterization of intrinsic properties of cingulate pyramidal neurons in adult mice after nerve injury Mol Pain 2009 5 73 10.1186/1744-8069-5-73 20015370 PMC2807858

[B34] Zhao MG Ko SW Wu LJ Toyoda H Xu H Quan J Li J Jia Y Ren M Xu ZC Zhuo M Enhanced presynaptic neurotransmitter release in the anterior cingulate cortex of mice with chronic pain J Neurosci 2006 26 8923 8930 10.1523/JNEUROSCI.2103-06.2006 16943548 PMC6675332

[B35] Bie B Brown DL Naguib M Increased synaptic GluR1 subunits in the anterior cingulate cortex of rats with peripheral inflammation Eur J Pharmacol 653 26 31 10.1016/j.ejphar.2010.11.027 21147092

[B36] Xu H Wu LJ Wang H Zhang X Vadakkan KI Kim SS Steenland HW Zhuo M Presynaptic and postsynaptic amplifications of neuropathic pain in the anterior cingulate cortex J Neurosci 2008 28 7445 7453 10.1523/JNEUROSCI.1812-08.2008 18632948 PMC3844787

[B37] Li XY Ko HG Chen T Descalzi G Koga K Wang H Kim SS Shang Y Kwak C Park SW Shim J Lee K Collingridge GL Kaang BK Zhuo M Alleviating neuropathic pain hypersensitivity by inhibiting PKMzeta in the anterior cingulate cortex Science 330 1400 1404 10.1126/science.1191792 21127255

[B38] Geiger JR Melcher T Koh DS Sakmann B Seeburg PH Jonas P Monyer H Relative abundance of subunit mRNAs determines gating and Ca2+ permeability of AMPA receptors in principal neurons and interneurons in rat CNS Neuron 1995 15 193 204 10.1016/0896-6273(95)90076-4 7619522

[B39] Gu JG Albuquerque C Lee CJ MacDermott AB Synaptic strengthening through activation of Ca2+-permeable AMPA receptors Nature 1996 381 793 796 10.1038/381793a0 8657283

[B40] Washburn MS Numberger M Zhang S Dingledine R Differential dependence on GluR2 expression of three characteristic features of AMPA receptors J Neurosci 1997 17 9393 9406 9390995 10.1523/JNEUROSCI.17-24-09393.1997 PMC6573423

[B41] Washburn MS Dingledine R Block of alpha-amino-3-hydroxy-5-methyl-4-isoxazolepropionic acid (AMPA) receptors by polyamines and polyamine toxins J Pharmacol Exp Ther 1996 278 669 678 8768718

[B42] Wei F Wang GD Kerchner GA Kim SJ Xu HM Chen ZF Zhuo M Genetic enhancement of inflammatory pain by forebrain NR2B overexpression Nat Neurosci 2001 4 164 169 10.1038/83993 11175877

[B43] Zhuo M Plasticity of NMDA receptor NR2B subunit in memory and chronic pain Mol Brain 2009 2 4 10.1186/1756-6606-2-4 19192303 PMC2644299

[B44] Wu LJ Toyoda H Zhao MG Lee YS Tang J Ko SW Jia YH Shum FW Zerbinatti CV Bu G Wei F Xu TL Muglia LJ Chen ZF Auberson YP Kaang BK Zhuo M Upregulation of forebrain NMDA NR2B receptors contributes to behavioral sensitization after inflammation J Neurosci 2005 25 11107 11116 10.1523/JNEUROSCI.1678-05.2005 16319310 PMC6725642

[B45] Chen L Liu JC Zhang XN Guo YY Xu ZH Cao W Sun XL Sun WJ Zhao MG Down-regulation of NR2B receptors partially contributes to analgesic effects of Gentiopicroside in persistent inflammatory pain Neuropharmacology 2008 54 1175 1181 10.1016/j.neuropharm.2008.03.007 18410946

[B46] Bird GC Lash LL Han JS Zou X Willis WD Neugebauer V Protein kinase A-dependent enhanced NMDA receptor function in pain-related synaptic plasticity in rat amygdala neurones J Physiol 2005 564 907 921 10.1113/jphysiol.2005.084780 15760935 PMC1464474

[B47] Li XY Ko HG Chen T Collingridge GL Kaang BK Zhuo M Erasing injury-related cortical synaptic potentiation as a new treatment for chronic pain J Mol Med10.1007/s00109-011-0768-9 21584648

[B48] Li P Zhuo M Silent glutamatergic synapses and nociception in mammalian spinal cord Nature 1998 393 695 698 10.1038/31496 9641681

[B49] Li P Kerchner GA Sala C Wei F Huettner JE Sheng M Zhuo M AMPA receptor-PDZ interactions in facilitation of spinal sensory synapses Nat Neurosci 1999 2 972 977 10.1038/14771 10526335

[B50] Woolf CJ Salter MW Neuronal plasticity: increasing the gain in pain Science 2000 288 1765 1769 10.1126/science.288.5472.1765 10846153

[B51] Esteban JA Shi SH Wilson C Nuriya M Huganir RL Malinow R PKA phosphorylation of AMPA receptor subunits controls synaptic trafficking underlying plasticity Nat Neurosci 2003 6 136 143 10.1038/nn997 12536214

[B52] Lee HK Takamiya K Han JS Man H Kim CH Rumbaugh G Yu S Ding L He C Petralia RS Wenthold RJ Gallagher M Huganir RL Phosphorylation of the AMPA receptor GluR1 subunit is required for synaptic plasticity and retention of spatial memory Cell 2003 112 631 643 10.1016/S0092-8674(03)00122-3 12628184

[B53] Vanhoose AM Clements JM Winder DG Novel blockade of protein kinase A-mediated phosphorylation of AMPA receptors J Neurosci 2006 26 1138 1145 10.1523/JNEUROSCI.3572-05.2006 16436600 PMC6674559

[B54] Wei F Zhuo M Potentiation of sensory responses in the anterior cingulate cortex following digit amputation in the anaesthetised rat J Physiol 2001 532 823 833 10.1111/j.1469-7793.2001.0823e.x 11313449 PMC2278568

[B55] Wu MF Pang ZP Zhuo M Xu ZC Prolonged membrane potential depolarization in cingulate pyramidal cells after digit amputation in adult rats Mol Pain 2005 1 23 10.1186/1744-8069-1-23 16111486 PMC1198253

[B56] Kandel ER The molecular biology of memory storage: a dialogue between genes and synapses Science 2001 294 1030 1038 10.1126/science.1067020 11691980

[B57] Rodriguez-Raecke R Niemeier A Ihle K Ruether W May A Brain gray matter decrease in chronic pain is the consequence and not the cause of pain J Neurosci 2009 29 13746 13750 10.1523/JNEUROSCI.3687-09.2009 19889986 PMC6666725

[B58] Kim SJ Thomas KS Calejesan AA Zhuo M Macromolecular synthesis contributes to nociceptive response to subcutaneous formalin injection in mice Neuropharmacology 1998 37 1091 1093 10.1016/S0028-3908(98)00099-9 9833638

[B59] Florence SL Taub HB Kaas JH Large-scale sprouting of cortical connections after peripheral injury in adult macaque monkeys Science 1998 282 1117 1121 9804549 10.1126/science.282.5391.1117

[B60] Metz AE Yau HJ Centeno MV Apkarian AV Martina M Morphological and functional reorganization of rat medial prefrontal cortex in neuropathic pain Proc Natl Acad Sci USA 2009 106 2423 2428 10.1073/pnas.0809897106 19171885 PMC2650172

[B61] Neugebauer V Li W Differential sensitization of amygdala neurons to afferent inputs in a model of arthritic pain J Neurophysiol 2003 89 716 727 12574449 10.1152/jn.00799.2002

[B62] Koga K Li X Chen T Steenland HW Descalzi G Zhuo M In vivo whole-cell patch-clamp recording of sensory synaptic responses of cingulate pyramidal neurons to noxious mechanical stimuli in adult mice Mol Pain 6 62 10.1186/1744-8069-6-62 PMC2954916 20920185

[B63] Becerra L Chang PC Bishop J Borsook D CNS activation maps in awake rats exposed to thermal stimuli to the dorsum of the hindpaw Neuroimage 54 1355 1366 10.1016/j.neuroimage.2010.08.056 20817102

